# Identification of drugs that restore primary cilium expression in cancer cells

**DOI:** 10.18632/oncotarget.7198

**Published:** 2016-02-04

**Authors:** Niamat Ali Khan, Nicolas Willemarck, Ali Talebi, Arnaud Marchand, Maria Mercedes Binda, Jonas Dehairs, Natalia Rueda-Rincon, Veerle W. Daniels, Muralidhararao Bagadi, Deepak Balaji Thimiri Govinda Raj, Frank Vanderhoydonc, Sebastian Munck, Patrick Chaltin, Johannes V. Swinnen

**Affiliations:** ^1^ KU Leuven - University of Leuven, Department of Oncology, Laboratory of Lipid Metabolism and Cancer, 3000 Leuven, Belgium; ^2^ Cistim Leuven vzw, Bioincubator 2, 3001 Leuven, Belgium; ^3^ European Molecular Biology Laboratory (EMBL), Grenoble Outstation and Unit of Virus Host-Cell Interactions (UVHCI), UJF-EMBL-CNRS, CS 90181, France; ^4^ VIB Bio Imaging Core and Center for the Biology of Disease, 3000 Leuven, Belgium; ^5^ KU Leuven - University of Leuven, Center for Human Genetics, 3000 Leuven, Belgium; ^6^ Centre for Drug Design and Discovery (CD3) KU Leuven R & D, Bioincubator 2, 3001 Leuven, Belgium

**Keywords:** cilium, cancer, small molecules, therapeutics, high content analysis

## Abstract

The development of cancer is often accompanied by a loss of the primary cilium, a microtubule-based cellular protrusion that functions as a cellular antenna and that puts a break on cell proliferation. Hence, restoration of the primary cilium in cancer cells may represent a novel promising approach to attenuate tumor growth. Using a high content analysis-based approach we screened a library of clinically evaluated compounds and marketed drugs for their ability to restore primary cilium expression in pancreatic ductal cancer cells. A diverse set of 118 compounds stimulating cilium expression was identified. These included glucocorticoids, fibrates and other nuclear receptor modulators, neurotransmitter regulators, ion channel modulators, tyrosine kinase inhibitors, DNA gyrase/topoisomerase inhibitors, antibacterial compounds, protein inhibitors, microtubule modulators, and COX inhibitors. Certain compounds also dramatically affected the length of the cilium. For a selection of compounds (Clofibrate, Gefitinib, Sirolimus, Imexon and Dexamethasone) their ability to restore ciliogenesis was confirmed in a panel of human cancer cell line models representing different cancer types (pancreas, lung, kidney, breast). Most compounds attenuated cell proliferation, at least in part through induction of the primary cilium, as demonstrated by cilium removal using chloral hydrate. These findings reveal that several commonly used drugs restore ciliogenesis in cancer cells, and warrant further investigation of their antineoplastic properties.

## INTRODUCTION

The primary cilium is a single, microtubule-based structure that protrudes from the surface of most mammalian cells [1]. It functions as a cellular antenna that captures signals from the environment and serves as a hub of key developmental and homeostatic signaling pathways including Wnt, planar cell polarity, and Hedgehog signaling [2–4]. Defects that compromise ciliary function contribute to specific disorders including Polycystic Kidney Disease (PKD), Birt-Hogg-Dubé (BHD) syndrome, Bardet-Biedl Syndrome (BBS) and others. Interestingly, several of these syndromes predispose affected carriers to the onset of cancer [5–7]. Moreover, an ever-increasing number of papers report on a decrease, loss, or distortion of the primary cilium in a variety of cancer types [8–11]. These include pancreatic cancer, breast cancer, melanoma, and prostate cancer. It is commonly assumed that the cilium puts a break on cell proliferation as it uses the same structural components required for chromosome segregation [12–14]. Loss of the cilium in cancer cells may, therefore, release this break and, moreover, may contribute to distorted cellular signaling, which is a hallmark of cancer. Hence, restoration of the primary cilium in cancer cells may represent a novel promising approach to attenuate cell proliferation and may provide novel opportunities for therapeutic antineoplastic intervention. However, so far few chemical compounds are known that normalize ciliogenesis in cancer cells.

Here, we have established a high content analysis-based screening method to identify small molecules that have the ability to restore the primary cilium in cancer cells. Further application of this method to screen a repurposing library composed of clinically evaluated compounds and marketed drugs revealed that many commonly used drugs restore the primary cilium in cancer cells and attenuate cell proliferation. These findings provide new insight into the spectrum of action of some commonly used drugs and may promote the expedited application of cilia-based therapies via repurposing of existing drugs in the field of oncology and beyond.

## RESULTS

### High content analysis-based screening for cilium-inducing compounds in pancreatic ductal cancer cells

**Figure 1:**
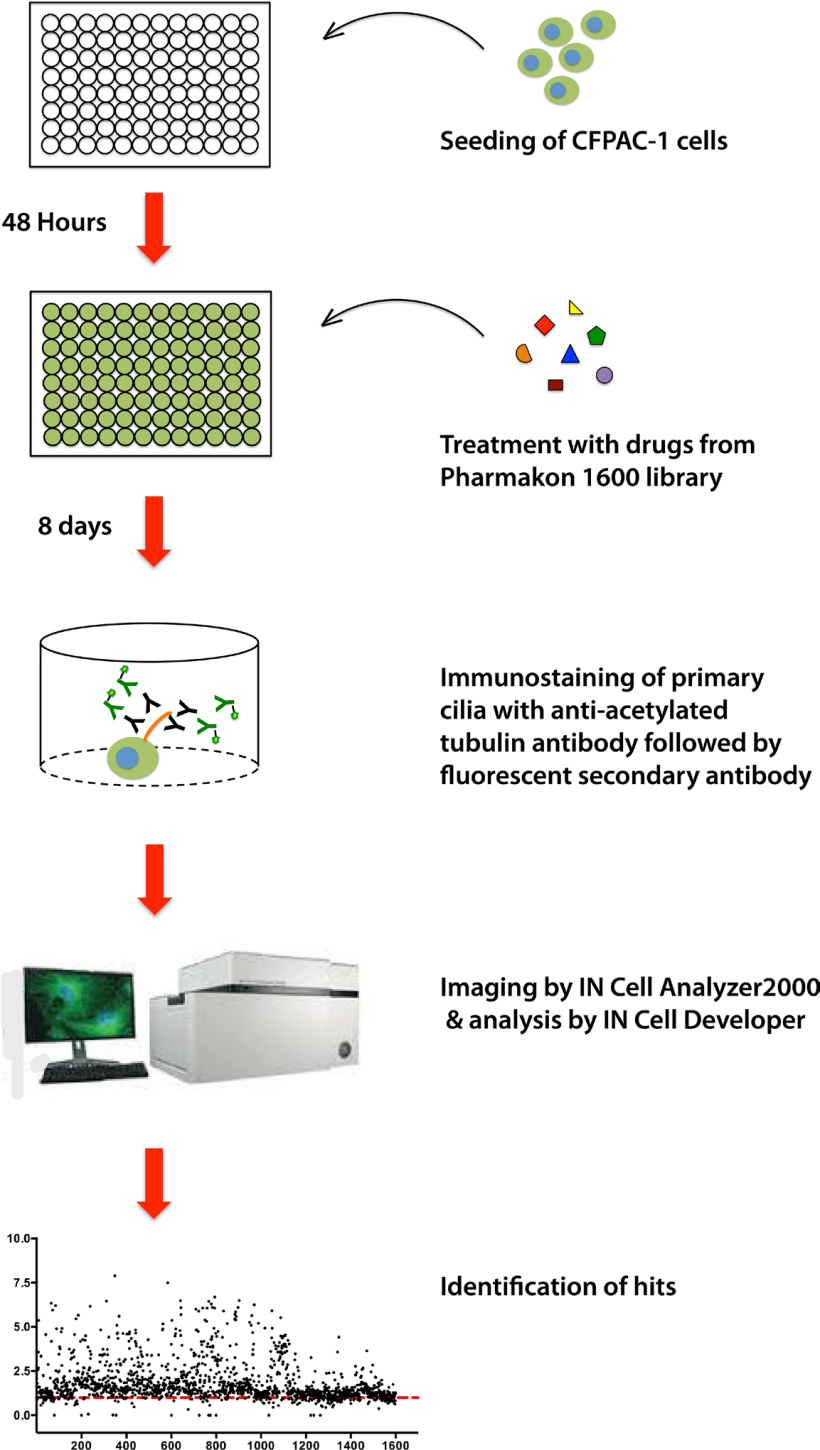
Schematic representation of the screening strategy of the Pharmakon Library using the human CFPAC-1 pancreatic cancer cell line model

**Figure 2:**
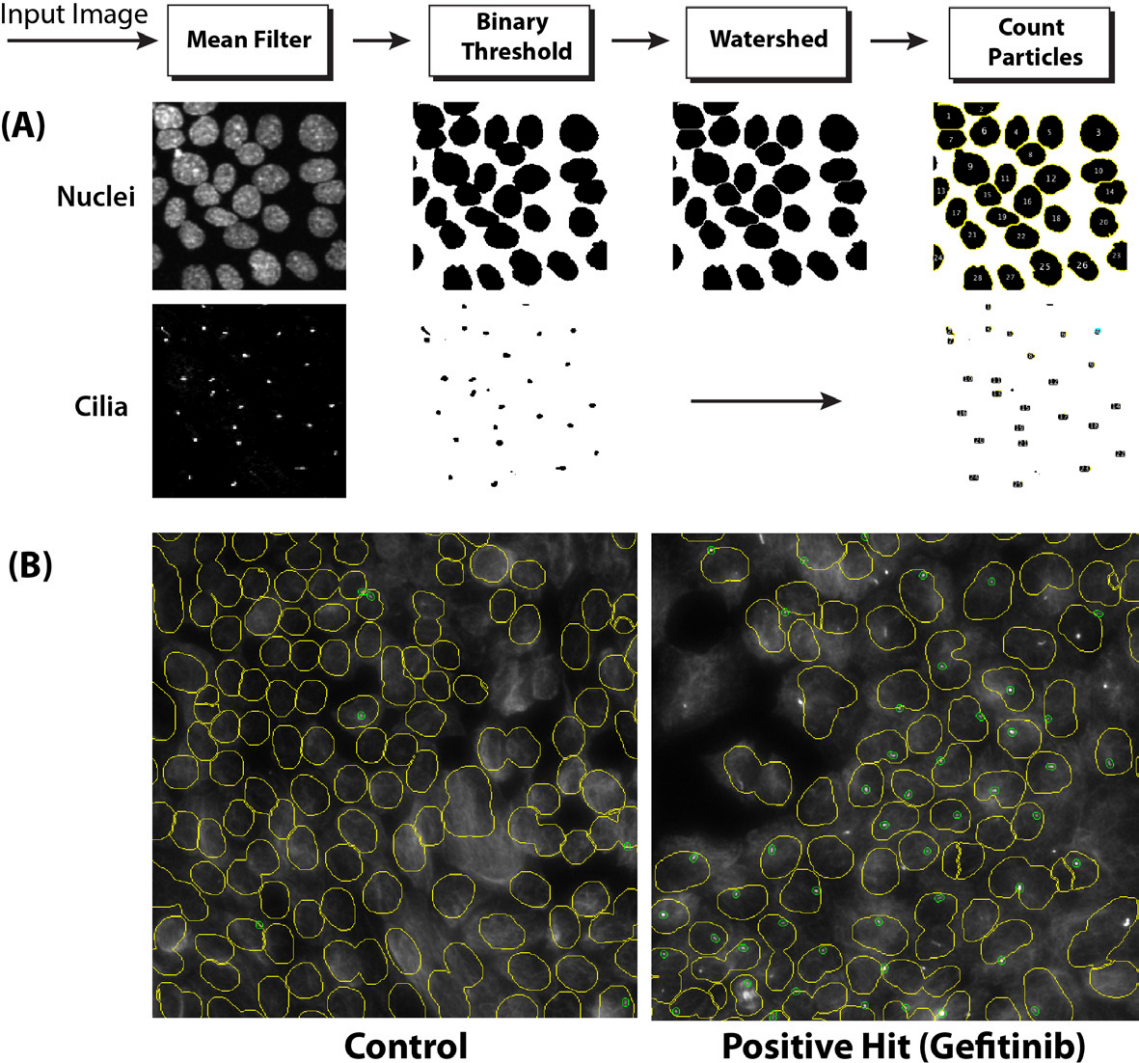
Imaging of ciliated cells using the IN Cell Analyzer 2000 Imaging system (**A**) Depiction of the image processing procedure, including the use of a watershed clump breaking algorithm to delineate nuclei. (**B**) Example of processed IN Cell Analyzer images of poorly ciliated control CFPAC-1 cells (left panel) and well-ciliated Gefitinib-treated CFPAC-1 cells (right panel). Nuclei are delineated by yellow lines. Cilia are indicated by small green circles.

**Figure 3:**
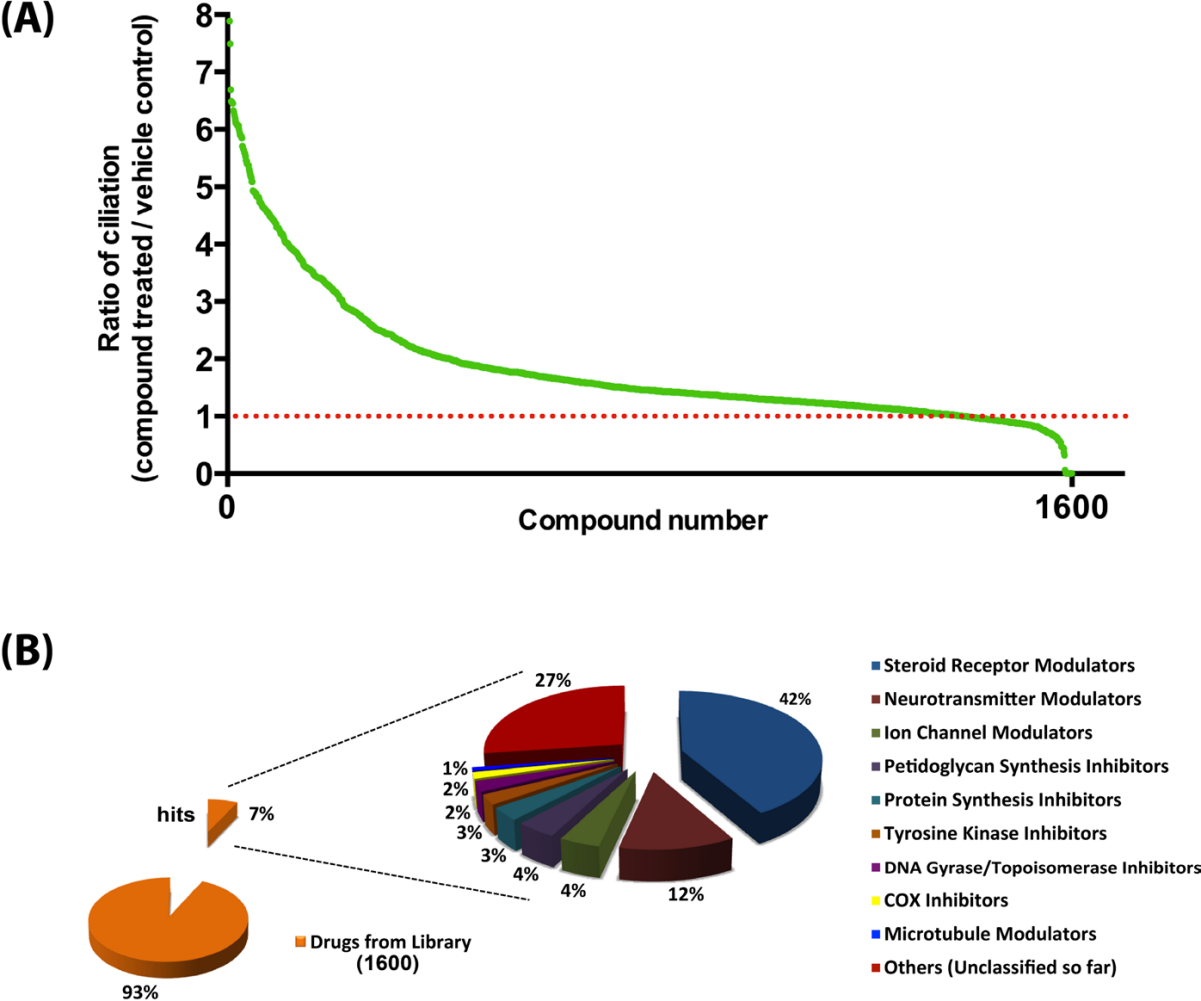
Summary of the outcome of the high content screen (**A**) Ciliogenic capacity of 1600 compounds of the Pharmakon 1600 library in CFPAC-1 cells using IN Cell Analyzer high content analysis. Compounds are ranked according to their potency to increase the percentage of ciliated cells relative to vehicle-treated cells (red dotted line). (**B**) Target diversity of confirmed hits is shown as a percentage of abundances of compounds in each class.

To screen a compound library for potential modulators of ciliogenesis, we developed an immunofluorescence microscopy-based phenotypic screening strategy in a 96-well format using an IN Cell 2000 High Content Analyzer (Figure 1). Pancreatic ductal cancer cells were chosen as the prime biological model in view of their well-documented loss of the primary cilium [10]. From a preliminary screening of a panel of human pancreatic cancer cell lines, CFPAC-1 cells were selected for the compound screening based on their ability to grow as flat monolayers, which is a prerequisite for accurate automated image acquisition, and for their inherent low rate of ciliogenesis, even at a high confluence (Figure 2). After incubation with 1600 different compounds from the Pharmakon 1600 library (all drugs added at 10 micromolar in DMSO), the percentage of ciliated cells was assessed in single wells by fluorescence microscopy-based visualization of the primary cilium using antibodies targeting cilium-associated acetylated tubulin. Nuclei were stained with Hoechst-33258. Images were analyzed using IN Cell Developer software according to the workflow illustrated in Figure 2. A cell was considered ciliated when the primary cilium signal (fluorescent dot) was enclosed within the nuclear border of the segmented nuclei. If more than one cilium-like dot was detected within the nuclear border, the bigger dot was selected as the match. The number of ciliated cells was then determined by counting the total number of nuclei and linked cilia in 20 fields in a single well. Compounds were considered ciliogenic based on their ability to increase the percentage of ciliated cells by at least 3 standard deviations compared to vehicle control. Using this approach, 156 ciliogenic compounds were identified in the initial screen. To eliminate false positives, the initial hits were re-evaluated in triplicate in a secondary screen using the same screening strategy. In this screen 118 cilium-enhancing compounds were confirmed. 110 of these compounds were found to increase ciliogenesis by at least a factor 2 (Figure 3A). Besides cilium-enhancing compounds, we identified 22 compounds that decreased ciliogenesis by at least 2-folds. The stimulatory compounds were categorized into 9 different classes based on their potential molecular targets (Table 1). More than one third of the positive compounds (49/118) were classified as glucocorticoids, fibrates or other nuclear receptor modulators (Figure 3B). 14/118 compounds were categorized as neurotransmitter modulators. Other classes included ion channel modulators, tyrosine kinase inhibitors, DNA gyrase/topoisomerase inhibitors, antibacterial compounds, protein synthesis inhibitors, microtubule modulators, and COX inhibitors.

**Table 1:** List of ciliogenic drugs identified from the Pharmakon 1600 library screen

DRUG NAME	CILIATION RATIO	ACTION	TARGET
***1. STEROID RECEPTOR MODULATORS***			
Glucocorticoid receptor modulators			
HYDROCORTISONE BUTYRATE	6.33	Glucocorticoid, anti-inflammatory	GR
AMCINONIDE	6.30	Glucocorticoid, anti-inflammatory	GR
DESONIDE	6.25	Anti-inflammatory, glucocorticoid	GR
CLOBETASOL PROPIONATE	6.11	Glucocorticoid, anti-inflammatory	GR
PREDNISOLONE HEMISUCCINATE	6.10	Anti-inflammatory, glucocorticoid	GR
BETAMETHASONE 17, 21-DIPROPIONATE	6.00	Glucocorticoid, anti-inflammatory	GR
ALCLOMETAZONE DIPROPIONATE	5.89	Anti-inflammatory, glucocorticoid	GR
DESOXYMETASONE	5.64	Anti-inflammatory	GR
BETAMETHASONE ACETATE	5.26	Anti-inflammatory	GR
PREDNICARBATE	5.19	Anti-inflammatory, glucocorticoid	GR
TRIAMCINOLONE DIACETATE	5.16	Anti-inflammatory	GR
DEXAMETHASONE	5.09	Glucocorticoid	GR
PREDNISOLONE SODIUM PHOSPHATE	5.09	Anti-inflammatory, glucocorticoid	GR
METHYLPREDNISOLONE SODIUM SUCCINATE	4.93	Glucocorticoid, anti-inflammatory	GR
TRIAMCINOLONE ACETONIDE	4.88	Anti-inflammatory	GR
BECLOMETHASONE DIPROPIONATE	4.83	Anti-asthmatic, topical antiinflammatory	GR
TRIAMCINOLONE	4.82	Glucocorticoid	GR
HYDROCORTISONE HEMISUCCINATE	4.82	Glucocorticoid	GR
FLUOROMETHOLONE	4.71	Glucocorticoid, Anti-inflammatory	GR
ISOFLUPREDNONE ACETATE	4.68	Anti-inflammatory	GR
DEXAMETHASONE SODIUM PHOSPHATE	4.66	Glucocorticoid, Anti-inflammatory	GR
FLUOCINOLONE ACETONIDE	4.65	Glucocorticoid, Anti-inflammatory	GR
HYDROCORTISONE PHOSPHATE TRIETHYLAMINE	4.64	Glucocorticoid	GR
DEFLAZACORT	4.62	Anti-inflammatory	GR
BUDESONIDE	4.61	Anti-inflammatory	GR
DEXAMETHASONE ACETATE	4.60	Glucocorticoid, Anti-inflammatory	GR
FLUMETHAZONE PIVALATE	4.59	Glucocorticoid, Anti-inflammatory	GR
PREDNISOLONE ACETATE	4.28	Glucocorticoid	GR
FLUDROCORTISONE ACETATE	4.20	Mineralocorticoid	GR/MR
PREDNISOLONE	4.27	Glucocorticoid	GR
HYDROCORTISONE ACETATE	4.18	Glucocorticoid, antiinflammatory	GR
FLUTICASONE PROPIONATE	4.12	Anti-inflammatory	GR
FLUNISOLIDE	3.87	Anti-inflammatory	GR
FLUMETHASONE	3.82	Anti-inflammatory	GR
FLUOCINONIDE	3.78	Anti-inflammatory, Glucocorticoid	GR/MR
DIFLORASONE DIACETATE	3.74	Anti-inflammatory, glucocorticoid	GR
BETAMETHASONE SODIUM PHOSPHATE	3.62	Anti-inflammatory, glucocorticoid	GR
BETAMETHASONE VALERATE	3.56	Glucocorticoid	GR
MEDRYSONE	3.54	Glucocorticoid	GR
HYDROCORTISONE VALERATE	3.52	Anti-inflammatory, glucocorticoid	GR
FLURANDRENOLIDE	3.45	Anti-inflammatory	GR
BETAMETHASONE	3.40	Glucocorticoid, anti-inflammatory	GR
METHYLPREDNISOLONE	3.34	Glucocorticoid	GR
**Estrogen receptor modulators**			
ESTRADIOL BENZOATE	3.96	Estrogen	ER
**Progesteron receptor modulators**			
MEDROXYPROGESTERONE ACETATE	3.42	Contraceptive	PR agonist
**Thyroid receptor modulators**			
IOPANIC ACID	3.29	Radio-opaque agent	5′deiodinase 73–74 conversion
LIOTHYRONINE	1.35	Thyroid hormone blocker, antidepressant	THR
**PPAR receptor modulators**			
CLOFIBRATE	6.06	Antihyperlipidemic	(PPAR) alpha agonist
PIOGLITAZONE HYDROCHLORIDE	2.15	Antidiabetic	(PPAR) gamma agonist
***2. NEUROTRANSMITTER MODULATORS***			
**Serotonin transporter inhibitors**			
PAROXETINE HYDROCHLORIDE	6.69	Antidepressant	Serotonin transporter
MILNACIPRAN HYDROCHLORIDE	3.80	Inhibitor of norepinephrine and seritonin uptake, treatment of fibromyalgia	Serotonin transporter			
**Serotonin breakdown inhibitors**			
PARGYLINE HYDROCHLORIDE	4.55	Antihypertensive	MAO
**Serotonin receptor inhibitors**			
TEGASEROD MALEATE	5.90	5HT4 receptor agonist, peristaltic stimulant	5HT receptor
ALMOTRIPTAN	3.96	5HT 1B/2D receptor agonist	5HT receptor
RIZATRIPTAN BENZOATE	1.45	5HT-1B/1D agonist, antimigraine	5HT receptor
**Dopamine receptor modulators**			
PERGOLIDE MESYLATE	3.59	Dopamine receptor agonist	DR
**Nicotinic cholinergic receptor modulators**			
GALLAMINE TRIETHIODIDE	2.27	Muscle relaxant (skeletal)	NCR antagonist
**Mu-opiod receptor modulators**			
LOPERAMIDE HYDROCHLORIDE	3.85	Ca channel blocker	MOR agonist
**Alpha1-adrenergic receptor modulators**			
OXYMETAZOLINE HYDROCHLORIDE	3.55	Adrenergic agonist, nasal decongestant	A1AR agonist
ADRENOLONE HYDROCHLORIDE	2.42	Adrenergic (opthalmic)	A1AR agonist
**Alpha2-adrenergic receptor modulators**			
XYLAZINE	3.76	Analgesic	A2AR
**Beta1-adrenergic receptor modulators**			
DOBUTAMINE HYDROCHLORIDE	4.83	Cardiotonic	B1AR agonist
ACEBUTOLOL HYDROCHLORIDE	3.90	Antihypertensive, antianginal, antiarrhythmic	B1AR antagonist
***3. ION CHANNEL MODULATORS***			
CAPSAICIN	4.52	Analgesic (topical)	TRPV1 channel
ESOMEPRAZOLE POTASSIUM	4.47	Gastric acid secretion inhibitor	H+/K+ exchange, alpha polypeptide
BUPIVACAINE HYDROCHLORIDE	4.29	Anesthetic (local)	SCN10A blocker
SURAMIN HEXASODIUM	2.85	Antiprotozoal, trypanocidal, antiviral	ATP-activated ion channel blocker
OXCARBAZEPINE	1.38	Antipsychotic / Na channel inhibition	SCN1A blocker
***4. COX INHIBITORS***			
KETOROLAC TROMETHAMINE	3.92	Anti-inflammatory / cyclooxygenase	COX
INDOPROFEN	1.88	Analgesic, anti-inflammatory / cyclooxygenase	COX
***5. TYROSINE KINASE INHIBITORS***			
GEFITINIB	4.89	Antineoplastic / EGFR inhibitor	TKR
LEFLUNOMIDE	4.24	Antineoplastic, PDGF receptor blocker	TKR
TANDUTINIB	2.81	Tyrosine kinase inhibitor	TKR
***6. ANTIBACTERIAL PEPTIDOGLYCAN SYNTHESIS INHIBITORS***			
CEFPROZIL	4.48	Antibacterial	Peptidoglycan synthesis
CEFOXITIN SODIUM	4.11	Antibacterial	Peptidoglycan synthesis
CEFAMANDOLE SODIUM	3.76	Antibacterial	Peptidoglycan synthesis
HETACILLIN POTASSIUM	1.90	Antibacterial	Peptidoglycan synthesis
CEFTIBUTEN	1.42	Antibacterial	Peptidoglycan synthesis
***7. DNA GYRASE/TOPOISOMERASE INHIBITORS***			
CIPROFLOXACIN	6.45	Antibacterial, fungicide	DNA gyrase topoisomerase
GEMIFLOXACIN MESYLATE	4.49	Antibacterial	DNA gyrase topoisomerase
LOMEFLOXACIN HYDROCHLORIDE	3.94	Antibacterial	DNA gyrase topoisomerase
***8. PROTEIN SYNTHESIS INHIBITORS***			
SIROLIMUS	6.06	Immunosuppressant, antineoplastic; rapamycin	mTOR / protein synthesis
OXYTETRACYCLINE	4.02	Antibacterial	Protein synthesis
MECLOCYCLINE SULFOSALICYLATE	3.57	Antibacterial	Protein synthesis
GENTAMICIN SULFATE	2.45	Antibacterial	Protein synthesis
***9. MICROTUBULE MODULATORS***			
DOCETAXEL	2.51	Antineoplastic	Microtubule
***OTHERS (unclassified so far)***			
IMEXON	5.39	Antineoplastic	
SEMUSTINE	5.31	Antineoplastic	
DENATONIUM BENZOATE	4.92	Denaturing agent, bitter principle	
TRANILAST	4.82	Antiallergic, mast cell degranulation inhibitor, angiogenesis blocker	
DICHLORISONE ACETATE	4.42	Antipruretic	
OSELTAMIVIR PHOSPHATE	4.38	Antiviral	Neuraminidase
DIBENZOTHIOPHENE	4.36	Keratolytic	
PENTAGASTRIN	4.29	Gastric secretion indicator	
RETINYL PALMITATE	4.05	Provitamin, antixerophthalamic	
SULCONAZOLE NITRATE	4.03	Antifungal	Sterol 14alpha-demethylase
DOCUSATE SODIUM	4.02	Stool softener	Anionic surfactant
BIFONAZOLE	3.93	Antifungal, calmodulin antagonist	Sterol 14alpha-demethylase
TRANDOLAPRIL	3.89	Antihypertensive, ACE inhibitor	ACE
PROCARBAZINE HYDROCHLORIDE	3.60	Antineoplastic	
MANNITOL	3.59	Diuretic, sweetener, diagnostic aid	
ARGININE HYDROCHLORIDE	3.32	Ammonia detoxicant, diagnostic aid	
TOLNAFTATE	3.31	Antifungal	Squalene epoxidase
PENTETIC ACID	3.18	Chelating agent, diagnostic aid	
AVOBENZONE	3.04	Sunscreen	
IODOQUINOL	2.99	Antiamebic	
PRILOCAINE HYDROCHLORIDE	2.93	Anesthetic (local)	
AMPYZINE SULFATE	2.88	CNS stimulant	
BENZOXIQUINE	2.71	Anti-infective	
PRASUGREL	2.69	Platelet aggregation inhibitor	
TRIENTINE HYDROCHLORIDE	2.60	Chelating agent	
PARAROSANILINE PAMOATE	2.56	Antihelminthic, antischistosomal	
NADIDE	2.24	Alcohol and narcotic antagonist	
ANEBROMPHENIRAMINE MALEATE	2.14	H1 antihistamine	
ISOTRETINON	2.12	Anti-acne, antineoplastic	
ANETHOLE	2.02	Expectorant, gastric stimulant, insecticide	
CLOFZAMINE	1.92	Antibacterial, antilepretic, antituberculosis	Guanine, PLA2, inhibits Smase
LITHIUM CITRATE	1.38	Antidepressant	

Compounds are classified according to their potential targets and arranged in descending order of ciliogenic potential in CFPAC-1 cells as assessed by IN Cell analysis (expressed as ratio of% of ciliated cells in treated cultures versus vehicle control) within each group. GR, glucocorticoid receptor; MR, mineralocorticoid receptor; ER, estrogen receptor; PR, progesterone receptor; THR, thyroid hormone receptor; MAO, mono amine oxidase; DR, dopamine receptor; NCR, nicotinic cholinergic receptor; MOR, mu-opioid receptor; A1AR, alpha 1 adrenergic receptor; A2AR, alpha 2 adrenergic receptor; B1AR, beta 1 adrenergic receptor; COX, cyclooxygenase; TKR, tyrosine kinase receptor; ACE, angiotensin-converting enzyme.

### Confirmation of ciliogenic ability of representative compounds by confocal microscopy

**Figure 4:**
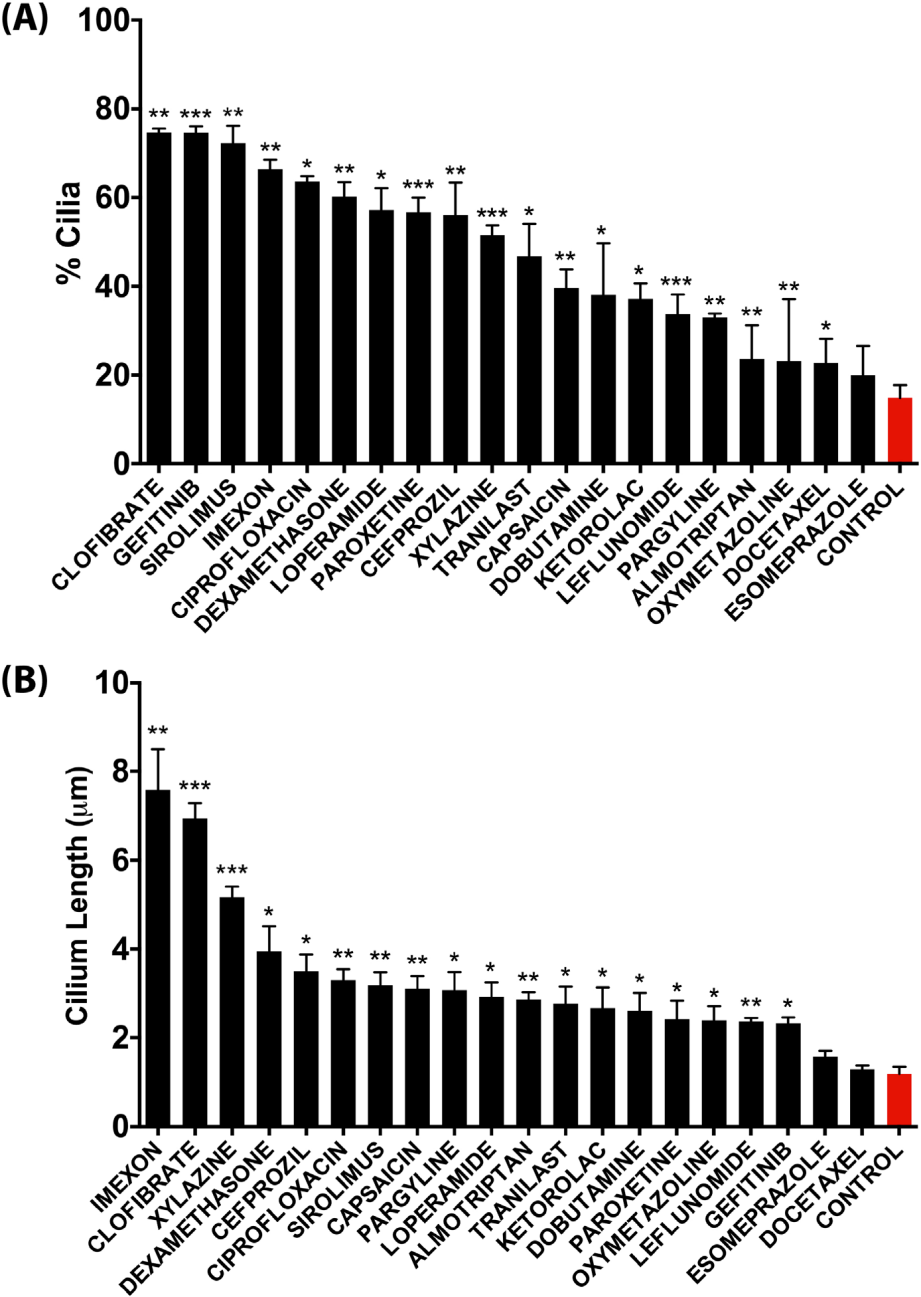
Quantitative analysis of the effect of representative compounds on the percentage of ciliation (**A**) and on average cilium length (**B**) in CFPAC-1 cells as assessed by confocal fluorescence microscopy analysis. Quantification was performed by counting 100–300 cells from at least three regions of the well. Data are presented as mean ± SEM, *p ≤ 0.05, **p ≤ 0.005, ***p ≤ 0.0005 as compared to control.

**Figure 5:**
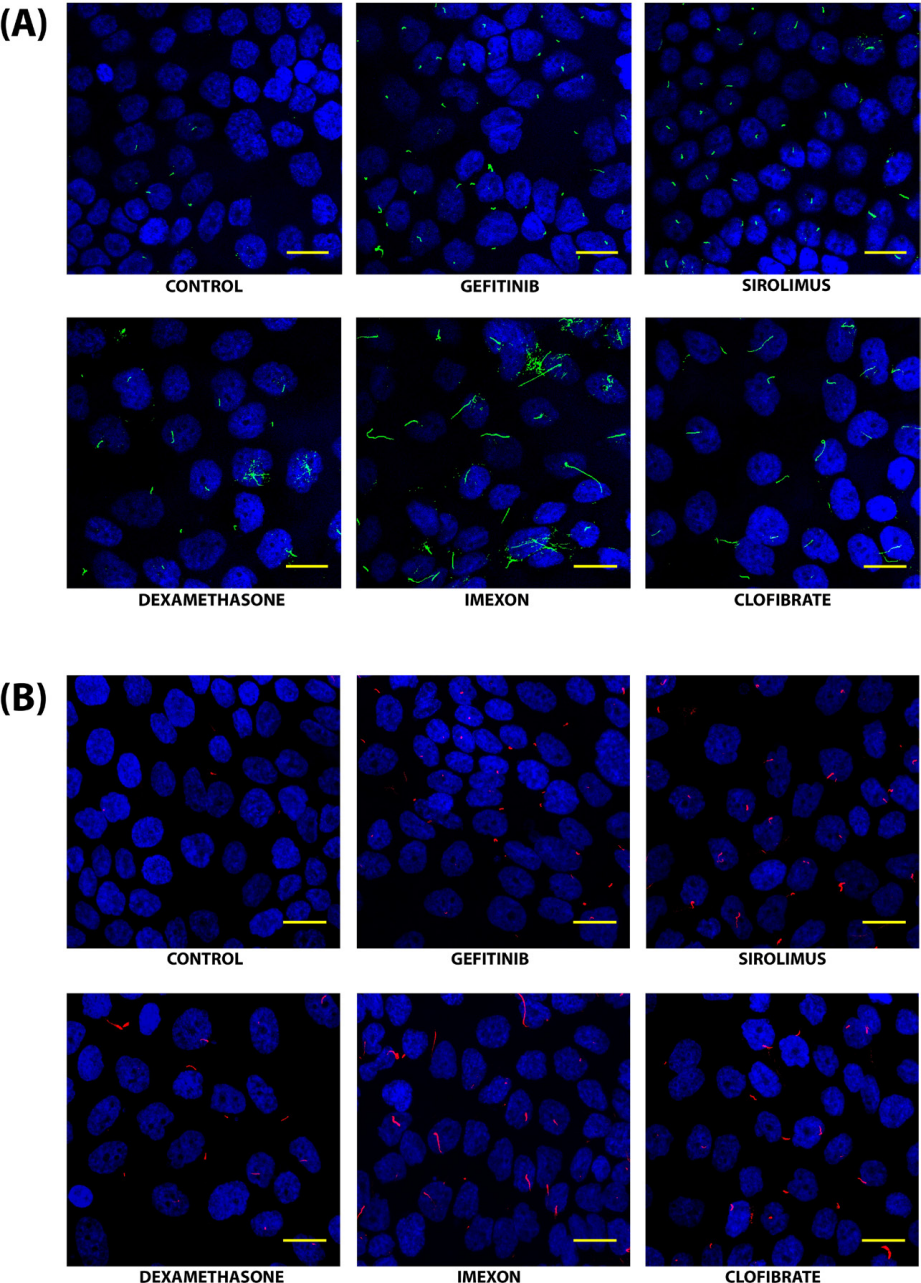
Confocal fluorescence microscopy images of primary cilia in CFPAC-1 cells treated with selected compounds Cilia were stained with an antibody against acetylated tubulin (green) (**A**) or with an antibody against IFT88 (red) (**B**). Nuclei were visualized by staining with DAPI (Blue). Images were captured using Bio-Rad Radiance confocal microscope through a 40X objective lens at 2.3X zoom. The scale bar represents 20 μm. Images were processed manually to optimally visualize cilia.

From the 118 cilium-enhancing compounds identified in the IN Cell Analyzer screen, we selected 20 representative compounds from the different classes for reconfirmation of their cilium modulating effect by a more robust confocal fluorescence microscopy approach, which also allowed assessment of changes in cilium length (Figure 4). Clofibrate, Gefitinib, Sirolimus, Imexon, Ciprofloxacin, and Dexamethasone were the most potent inducers of cilia in terms of percentage of ciliated cells, showing a statistically significant increase of 4-fold or more, relative to control vehicle-treated cells (Figure 4A). In terms of cilium length, Imexon, Clofibrate, and Xylazine induced a significant increase of 4-fold or more, relative to control conditions (Figure 4B). Some compounds like Clofibrate and Imexon were potent inducers of both cilium percentage and cilium length whereas other compounds primarily enhanced cilium percentage (e.g. Gefitinib). Figure 5A shows representative confocal microscopy pictures of primary ciliation of cells treated with the most potent compounds as revealed by staining for acetylated tubulin. To confirm that genuine primary cilia are induced by the drugs, cilia were stained for IFT88, an alternative marker of the primary cilium (Figure 5B).

### Identified ciliogenic drugs induce cilia in multiple cancer cell models

**Figure 6:**
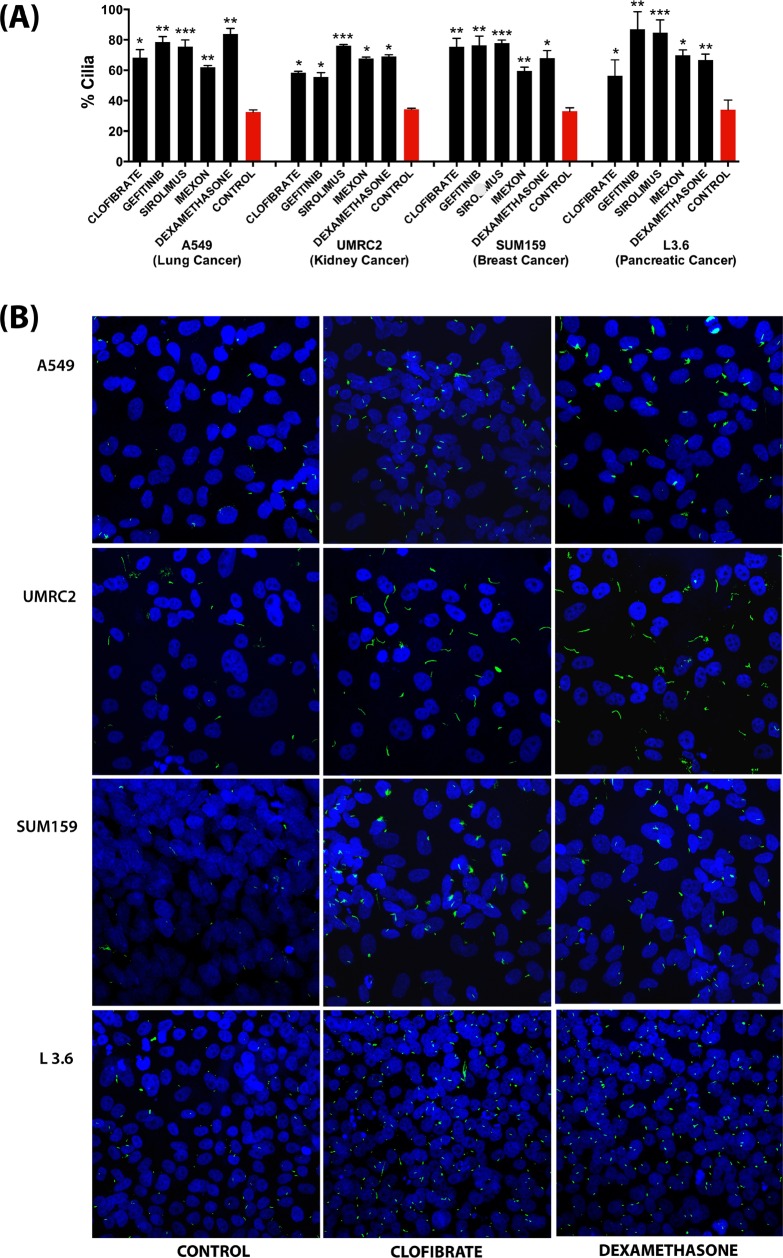
Effect of a selection of compounds on ciliogenesis in different cancer cell line models as assessed by confocal fluorescence microscopy analysis (**A**) Quantification of the percentage of ciliated cells. Data are presented as mean (*n =* 100–300) ± SEM, *p ≤ 0.05, **p ≤ 0.005, ***p ≤ 0.0005 as compared to control. (**B**) Representative images showing the effect of selected compounds on ciliation in different cancer cell line models. All Images were captured using Nikon C2 Eclipse Ti-E confocal microscope at 1.0X zoom using a 60x objective lens.

To corroborate the ability of these compounds to induce cilia in cancer cells, we tested a selection of the most potent compounds (Clofibrate, Gefitinib, Sirolimus, Imexon and Dexamethasone) in a panel of human cell lines representing different cancer types: A549 (lung cancer), UMRC2 (kidney cancer), SUM159 (breast cancer) and L3.6 (pancreatic cancer) cell lines. As shown in Figure 6, all compounds significantly increased the percentage of ciliated cells in all the four cell lines. These results confirm the potential of the identified compounds as cilium inducers in cancer cells.

### Ciliogenic drugs attenuate cell proliferation at least in part through induction of the primary cilium

**Figure 7:**
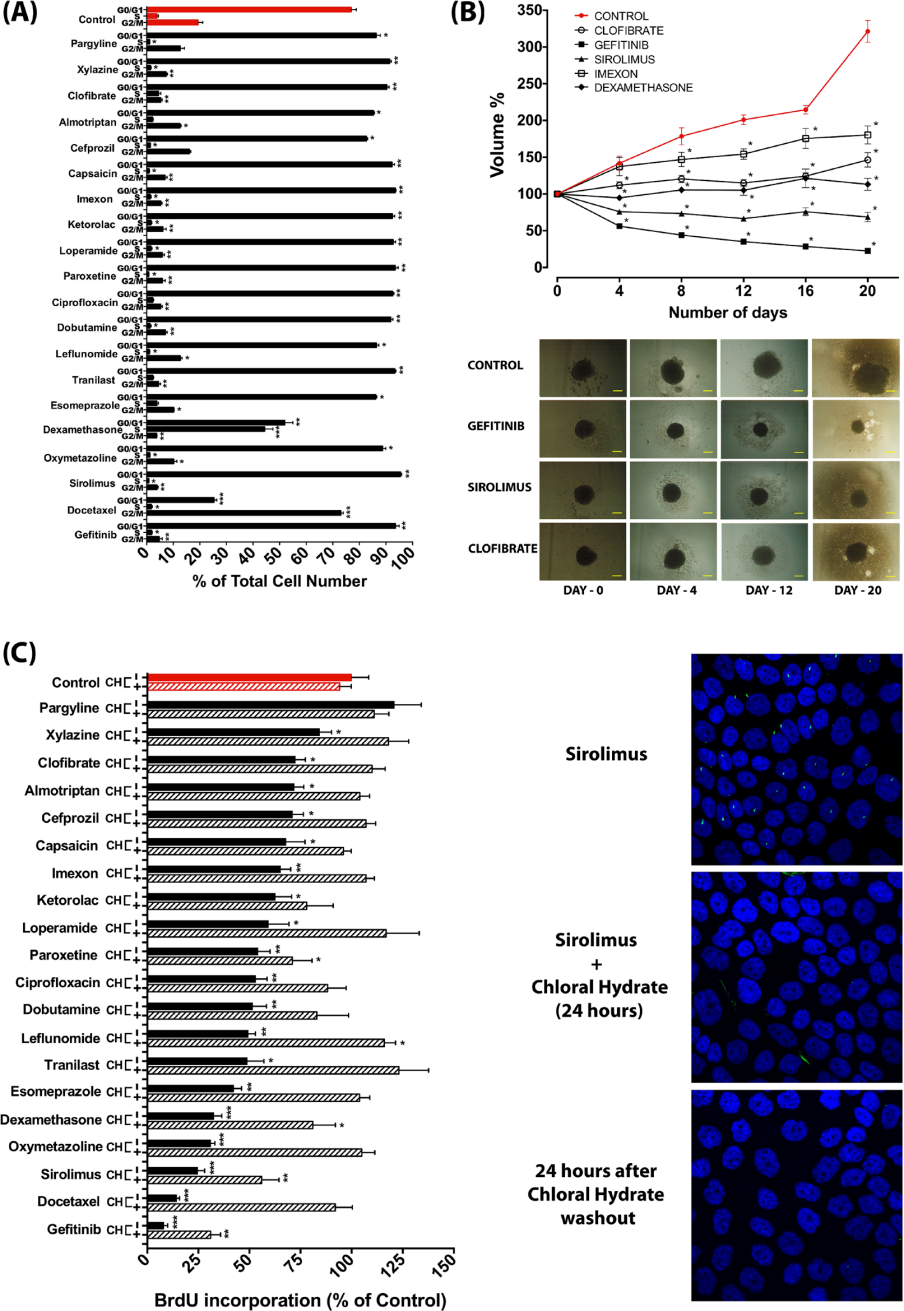
Anti-proliferative effect of ciliogenic compounds and involvement of the primary cilium (**A**) Changes in cell cycle profile as determined by FACS analysis of CFPAC-1 cells upon treatment with a selection of ciliogenic compounds. Data are presented as mean ± SEM, *p ≤ 0.05, **p ≤ 0.005, ***p ≤ 0.0005 as compared to control. (**B**) Effect of selected compounds on spheroid formation of L3.6 pancreatic cancer cells. Data are presented as mean of at least 6 spheroids ± SEM, *p < 0.0001 as compared to control. The bottom panel shows representative images of spheroids treated with vehicle control and with selected drugs. (**C**) Effect of compounds on cell proliferation of CFPAC-1 cells as measured by BrdU incorporation and impact of deciliation by treatment with chloral hydrate (CH). Proliferation of cells was measured by BrdU incorporation 24 hours after deciliation. Differences in proliferation were expressed as percentage of BrdU incorporation as compared to untreated control (red bar). Data are presented as mean ± SEM, *p ≤ 0.05, **p ≤ 0.005, ***p ≤ 0.0005. Confocal microscope images show the effect of 4 mM chloral hydrate on sirolimus-treated CFPAC-1 cells. Primary cilia are stained for acetylated tubulin (green) and nuclei are stained with DAPI (blue).

As the presence of the primary cilium is dependent on the cell cycle and is most prominent in the G0/G1 phase, we examined the effect of the selected drugs on the cell cycle using FACS analysis. Although under the culture condition that we used, cultures were not highly proliferative even in control conditions, most compounds resulted in a further increase in the percentage of cells in the G0/G1 phase, indicative of a further induction of growth arrest (Figure 7A). In line with these findings, most compounds attenuated cell proliferation as assessed by a spheroid formation assay of L3.6 cells (Figure 7B) and BrdU incorporation in CFPAC-1 cells (Figure 7C). To explore to what extent primary cilium occurs secondarily to the growth arrest or in fact actively contributes to the observed attenuation of cell proliferation, we assessed the effect of these compounds on cell proliferation in the presence of the deciliation agent chloral hydrate, which completely removed the cilium (Figure 7C). Interestingly, in most cases deciliation largely restored cell proliferation of compound-treated cells (Figure 7C), indicating that the antiproliferative effect of these compounds is at least in part caused by their ability to induce the primary cilium.

## DISCUSSION

Building on the emerging concept that loss of the primary cilium is linked to the development of several tumor types and that re-establishing the expression of this organelle may attenuate tumor growth, we have developed a semi-high-throughput approach to identify stimulators of ciliogenesis in cancer cells. Screening of the Pharmakon 1600 library resulted in the identification of 118 compounds that showed the ability to restore cilium expression in cancer cell line models. Among these were many glucocorticoids (43/118 hits or 43/47 glucocorticoids in the entire library). A diverse set of other drugs was identified including many neurotransmitter regulators and ion channel modulators. Some of the compounds from our screen such as dexamethasone, hydrocortisone, estradiol, pargyline and lithium have previously been described as enhancers of the percentage of ciliated cells and/or of cilium length [15–19]. Others, like beclomethasone, fluticasone, flunisolide, mannitol and arginine are known to affect ciliary beat frequency (CBF), which in many cases varies with dosage and length of treatment [20–23]. It has been suggested that beta-adrenergic receptor modulators may have a therapeutic role in the treatment of Primary Cilia Dyskinesia (PCD) [24, 25]. Pioglitazone has been shown to have beneficial effects in the treatment of Polycystic Kidney Disease (PKD) [26]. Cilio-modulatory activities have also been attributed to several other compounds from our screen like triamcinolone, budesonide, paroxetine, oxymetazoline and docetaxel [23, 27–30]. Imexon, a potent enhancer of cilium length as shown in our results, is a known inducer of apoptosis and cell cycle arrest in pancreatic cancer cells [31]. For this reason it has been used in combination drug trials along with gemcitabine for the treatment of patients with advanced pancreatic cancer. The anti-diarrheal drug loperamide, a good inducer of cilia in our models, has been reported as a potential anti-tumor agent due to its ability to induce apoptosis [32].

**Figure 8:**
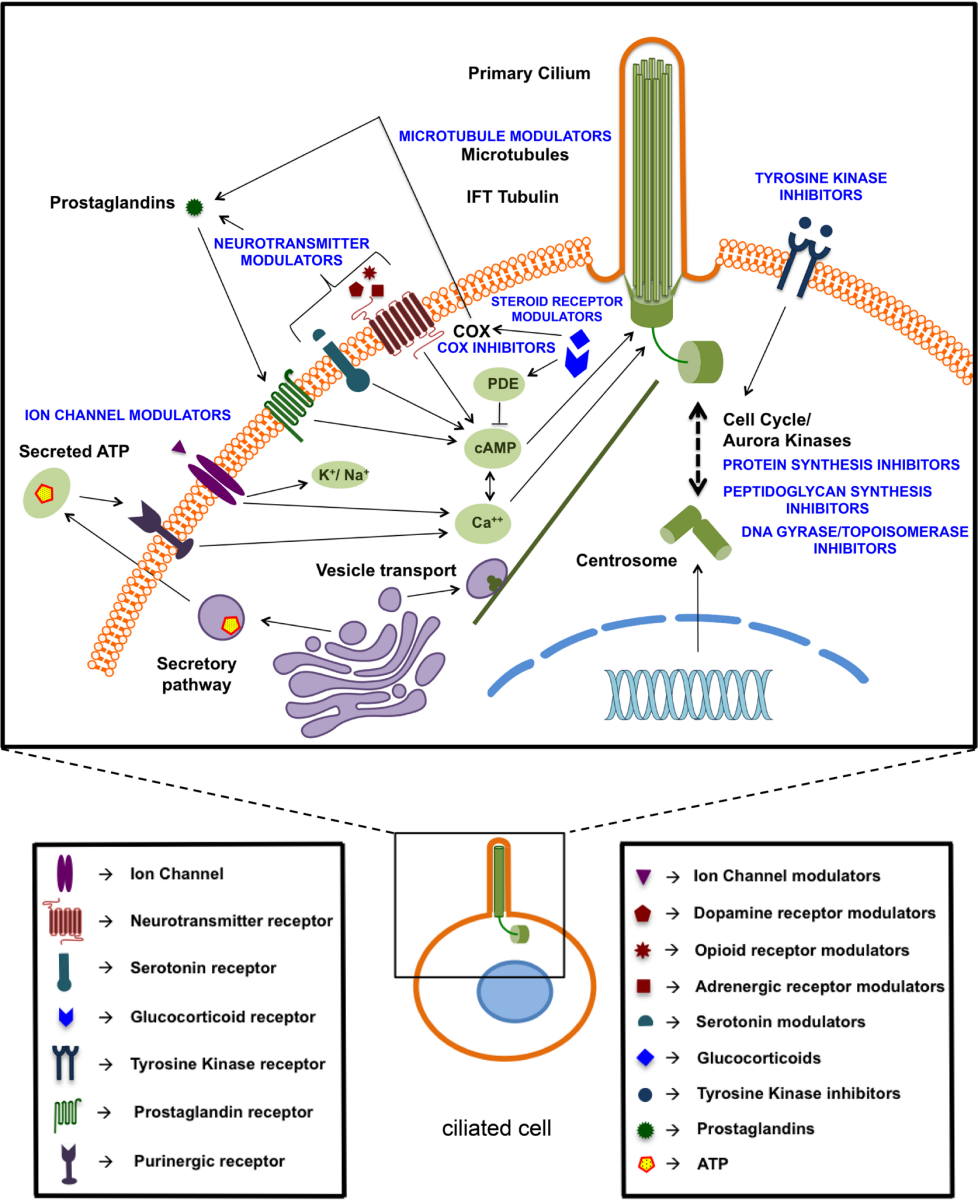
Schematic overview of identified ciliogenic compounds based on their potential targets or putative mechanism of action

With respect to the mechanisms underlying the cilium modulating effects of these compounds, many of them (steroid receptor modulators, neurotransmitter modulators, ion channel modulators) are known to affect levels of cAMP, calcium or other ions, which are established regulators of cilium expression and/or cilium length [33–35] (Figure 8). Microtubule modulators may affect the microtubule assembly and extension in the cilium or may disturb the transport towards the cilium [36–38]. Tyrosine kinase inhibitors may enhance ciliogenesis by inhibiting ligand-dependent activation of cell-cycle entry or by regulating the coordination of signaling events linked to cilium-centrosome axis, that control cell cycle, differentiation and migration [39, 40]. Ciliogenic effects of sirolimus (rapamycin) might be mediated through mTOR signaling which is known to modulate ciliary size and function through translational regulation [41]. Others (cephalosporins, DNA gyrase/topoisomerase inhibitors) are known to affect Aurora kinases [42–44], which are also well-established regulators of the primary cilium [45–47]. Several compounds may affect the cilium through extracellular ATP. Several studies show a relationship between intracellular calcium levels and extracellular ATP via the purinergic receptor [48–51]. Also glucocorticoids promote ATP release from the cell [52]. Many of these compounds also affect cell proliferation as known from the literature [31, 53–63] or as revealed in our spheroid and BrdU assays. This effect was not always proportional to the induction of ciliogenesis, which is not surprising given the various mechanisms of action of these compounds. The further characterization of these mechanisms may be challenging in part because of the incomplete understanding of the mechanisms of action of some of these drugs and the processes involved in ciliogenesis. Many of the compounds we identified cause cell cycle arrest in the G0/G1 phase, which is known to promote ciliogenesis [13], suggesting that the ciliogenic effect of some of these compounds may be indirect and may be downstream of cell cycle arrest. Nevertheless, our findings that deciliation of compound-treated cells restores cell proliferation strongly suggest that the compound-induced ciliogenesis plays an active role in the antiproliferative effects of these compounds.

Our findings that many common drugs have the ability to restore the primary cilium in cancer cell line models may provide interesting new insights in the spectrum of actions of these compounds and may warrant further investigation into application of some of these compounds in future antineoplastic approaches. They also promote the concept of harnessing the therapeutic potential of existing drugs for a novel use, generally referred to as drug repurposing [64–67]. Development of a new drug is a complex, time-consuming and costly process, mainly due to pharmacological hurdles like bioavailability, solubility, stability, toxicity etc., which form bottlenecks in the therapeutic development process. This can be overcome by drug repurposing, a strategy that reduces the time frame, decreases the costs and improves the success rate by redirecting existing drugs for a new indication. Most of the drugs from the Pharmakon 1600 Library have already been tested for safety in humans and data is available on their pharmacology, formulation and toxicity, paving the way for an accelerated development of cilia-based therapeutics. The success of our screening approach also sets the stage to screen other libraries of new compounds and to establish these as potential novel antineoplastic agents and/or agents exploitable in other cilium-related diseases, including classical ciliopathies.

## MATERIALS AND METHODS

### Cell lines

All cell lines were obtained from ATCC. A549 (lung cancer), UMRC2 (kidney cancer) and SUM159 (breast cancer) were maintained in DMEM medium (Life Technologies) supplemented with 10% FBS (Life Technologies). CFPAC-1 (pancreatic cancer) cells were cultured in RPMI-1640 medium (Life Technologies) supplemented with 10% FBS. L3.6 (pancreatic cancer) cells were grown in RPMI-1640 medium supplemented with 10% FBS and 2mM L-Glutamine (Life Technologies). All cell lines were incubated in a humidified incubator at a temperature of 37°C and 5% levels of CO_2_.

### Compound library screening

CFPAC-1 cells were seeded in 96-well microplates at a density of 10, 000 cells/well containing 200 μl culture medium supplemented with 10% FBS and incubated for 48 hours in a humidified incubator (37°C and 5% CO_2_), after which medium was refreshed with 200 μl of RPMI-1640 medium containing 2% FBS. The Pharmakon 1600 chemical library consisting of 1600 clinically evaluated compounds and marketed drugs was procured from MicroSource Discovery Systems Inc. (U.S.A). The compounds were dissolved as 10 mM stock solutions in DMSO and added to culture medium to a final concentration of 10 μM. Media and compounds were refreshed on the fourth day after the initial addition of the compounds. After 8 days of compound incubation, the cells were chemically fixed and stained with anti-acetylated tubulin antibody (Sigma, Cat No. T6793-.5ML), which stains the ciliary axoneme. A fluorescent secondary antibody (Life Technologies, AlexaFluor 488, Cat No. A21145) was used against the primary antibody. The nuclei were counterstained with Hoechst-33258 (Cat No: 382061, Calbiochem). 20 randomized fields per well were imaged at a single plane of focus at 20X magnification in DPBS (Sigma) to reduce auto-fluorescence from the medium and to minimize signal-to-noise ratio. Images acquired by the IN Cell Analyzer 2000 (GE Healthcare) were analyzed using IN Cell Developer software (GE Healthcare).

### Confocal microscopy

CFPAC-1 cells were seeded on glass coverslips in 12-well plates containing 1 ml of culture medium per well. At 30% confluency, cell media was replaced by low serum medium containing 2% FBS. After 8 days of compound treatment, cells were fixed with 4% Formaldehyde (Merck), permeabilized with 0.1% Triton X100 (Merck) in DPBS, blocked with 1% BSA (Applichem) in DPBS, and incubated with 1:1000 dilution of anti-acetylated tubulin antibody or 1:500 dilution of anti-IFT88 antibody (Cat. No. 13967–1-AP, Proteintech) for 1 h, followed by incubation with 1:1000 dilution of fluorescent secondary antibody for 1 h. Nuclei were counterstained with DAPI (Vector Laboratories, Vectashield (Cat. No. H-1500). Images of primary cilia were captured by acquiring Z-stacks using either a Bio-Rad Radiance or Nikon C2 Eclipse Ti-E confocal laser scanning microscope by 40X or 60X oil immersion lenses. All drugs selected for confocal reconfirmation experiments were purchased from Sigma, except for Almotriptan Malate and Sirolimus which were obtained from Selleckchem. Gefitinib was from Invivogen, Cefprozil Monohydrate from Abcam and Imexon from MicroSource Discovery Systems Inc.

### Confirmation of ciliogenesis in other cancer cell lines

A549, UMRC2, SUM159 and L3.6 cells were treated as indicated and images were acquired as previous. Image stacks captured by confocal microscope were processed and analyzed for cilia percentage and cilium length using ImageJ software.

### Chemical deciliation

CFPAC-1 cells treated with ciliogenic compounds were exposed to 4 mM chloral hydrate (Cat. No. C8383–100G, Sigma) for 24 h to remove cilia [68]. Following deciliation, chloral hydrate was washed out and the cells were allowed to grow in fresh culture medium for 24 h before BrdU addition and measurement of proliferation.

### Proliferation assays

Spheroid assay - Tumor spheroids were formed on agarose-coated (1%) 96-well plates by seeding L3.6 pancreatic cancer cells in 200 μl of culture medium. The plates were kept undisturbed in a humidified incubator at 37°C and 5% CO_2_ for 4 days to facilitate spheroid formation. Treatment with compounds was started when the cells aggregated to form spheroids. Compounds and 50% of the medium were refreshed every 4th day. Images were captured at the beginning of treatment with 5X objective mounted on an inverted light microscope. Imaging was done every 4 days for 20 days from the start of treatment. The images were analyzed by ImageJ software (rsb.info.nih.gov/ij) to calculate the differences in the volume of spheroids at different time points.

BrdU incorporation assay - BrdU incorporation assay was performed using 5-Bromo-2-deoxy-uridine Labeling and Detection Kit III (Cat. No. 11444611001, Roche) according to manufacturer›s instructions.

### Cell cycle analysis

CFPAC-1 cells were plated at a density of 300, 000 cells/well of a 6-well plate and allowed to grow for two days. This was followed by compound treatment for 48 h and harvesting of cells by trypsinization. Cells were fixed with 4% formaldehyde and permeabilized in 0.1% Triton X100 solution. The permeabilized cells were washed with PBS and stained with Vybrant DyeCycle Green Stain (Cat. No. V35004, ThermoFisher Scientific). Cell cycle data was acquired by measuring at least 10, 000 events per sample with BD FACSCanto Flow Cytometer (Becton Dickinson). Data was analyzed using FlowJo software (Becton Dickinson) and the cell cycle distribution was calculated by ImageJ software.

### Statistical analysis

GraphPad Prism version 6 for Mac OS X (GraphPad Software, San Diego, California, USA) was used for statistical analysis. All data are expressed as mean ± SEM. Differences between two groups were assessed using the *t*-test. To determine differences between more than two groups, two-way ANOVA was used followed by Dunnett's multiple comparisons test. Differences with *p* values of < 0.05 were considered to be statistically significant.
